# First description of B chromosomes in the *Hyphessobrycon* (Characiformes, Characidae) genus: a hypothesis for the extra element of *Hyphessobrycon eques* Steindachner, 1882

**DOI:** 10.3897/CompCytogen.v9i3.5224

**Published:** 2015-07-03

**Authors:** Diovani Piscor, Patricia Pasquali Parise-Maltempi

**Affiliations:** 1 Instituto de Biociências, Departamento de Biologia, Laboratório de Citogenética, Universidade Estadual Paulista “Júlio de Mesquita Filho” (UNESP), Av. 24A, 1515, CEP: 13506-900, Rio Claro, SP, Brazil

**Keywords:** Karyotype, supernumerary chromosomes, C-banding, heteromorphism, chromosome evolution

## Abstract

The *Hyphessobrycon* are allocated in the *incertae sedis* group of the Characidae family, one of the genera with more species of the group. The chromosomes of some species of *Hyphessobrycon* are known, and the diploid number most common for genus is 2n = 50 chromosomes. The aims of this study were to examine the karyotype macrostructure in the *Hyphessobrycon eques* Steindachner, 1882, and show a new origin hypothesis for B chromosomes. The diploid number observed for *Hyphessobrycon eques* was 2n = 52 chromosomes, and a karyotype formulae of 12m + 18sm + 8st + 14a, with FN (fundamental number) = 90 for both sexes. Only two females showed one B chromosome. The heterochromatin was observed mainly on centromeric regions, and in the long arm of the B chromosome. In this paper, the relationship of the B chromosome of *Hyphessobrycon eques* with an occasional chromosome rearrangement was discussed.

## Introduction

The *Hyphessobrycon* are allocated in the *incertae sedis* group of the Characidade family (Lima et al. 2003) with more than 130 species (e.g., Lima and Moreira 2003, Carvalho and Bertaco 2006). Among these, a species known as “Mato Grosso” has been considered *Hyphessobrycon callistus* (Boulenger, 1900) for a long time, however with the revision of Weitzman and Palmer (1997), it started to be classified as *Hyphessobrycon eques*.

The chromosomal data of the *Hyphessobrycon* genus are restricted primarily to the knowledge of the diploid number. Literature data showed that the diploid number vary between 2n = 42 and 52 chromosomes, being 2n = 50 chromosomes the most frequently observed number for the genus, i.e. *Hyphessobrycon scholzei* Ahl, 1937 (Arefjev 1990), *Hyphessobrycon reticulatus* Ellis, 1911 (Wlasiuk and Garcia 1996, Carvalho et al. 2002a), *Hyphessobrycon bifasciatus* Ellis, 1911 (Miyazawa 1997), Hyphessobrycon aff. santae Eigenmann, 1907 (Miyazawa 1997) and *Hyphessobrycon anisitsi* Eigenmann, 1907 (Centofante et al. 2003). According to Carvalho et al. (2002a) many species of the genus have a known chromosome set, though for many species only the haploid number has been described.

The B chromosomes have been described in many neotropical fish groups (see, for example, Maistro et al. 1992, Oliveira et al. 1997, Maistro et al. 2000, Torres-Mariano and Morelli 2008, Ferreira-Neto et al. 2012, Hashimoto et al. 2012, Silva et al. 2014). The occurrence of this type of chromosome among individuals of a population can be sporadic or commonly found for many individuals, and high frequency can be shown between them. It is also possible to find variations regarding to morphology, size, number and pattern of heterochromatin in the B chromosomes (Maistro et al. 1992, Venere et al. 1999, Cavallaro et al. 2000, Fernandes and Martins-Santos 2005, Artoni et al. 2006, Hashimoto et al. 2012, Barbosa et al. 2015).

Whereas the diversity of events described in an attempt to explain the origin and function of B chromosomes, the present study aims to demonstrate the probable origin of B chromosome in *Hyphessobrycon eques* through the study of heterochromatin, and describe for the first time the presence of an extra element in the *Hyphessobrycon* genus.

## Material and methods

The *Hyphessobrycon eques* (seven males and four females) specimens were obtained from Ribeirão Claro river (22°21'36"S, 47°30'42"W) in the state of São Paulo (SP), Brazil. The individuals were anesthetized with benzocaine (5%) and then used for cytogenetic analysis. The individuals were fixed in formaldehyde 10% and then in ethanol 70%, and placed in the ichthyological collection from Departamento de Biologia do Instituto de Biociências da UNESP, campus de Rio Claro. The chromosomes were obtained as described by Foresti et al. (1981). Chromosome morphologies were determined according to the ratio of the arms (the most frequently used classification system for fish chromosomes). Briefly, the length of the long arm (q) was divided by the length of the short arm (p) as cited by Piscor et al. (2013). Therefore, the chromosomes with two arms and an arm ratio (AR) of 1–1.7 were classified as metacentric (m), those with two arms and AR of 1.71–3 were classified as submetacentric (sm), and those with two arms and AR of 3.01–7 were classified as subtelocentric (st). Chromosomes with a single arm (AR >7) were considered to be acrocentric (a). Heterochromatin was observed using the C-band technique proposed by Sumner (1972).

## Results

**Figure 1.**
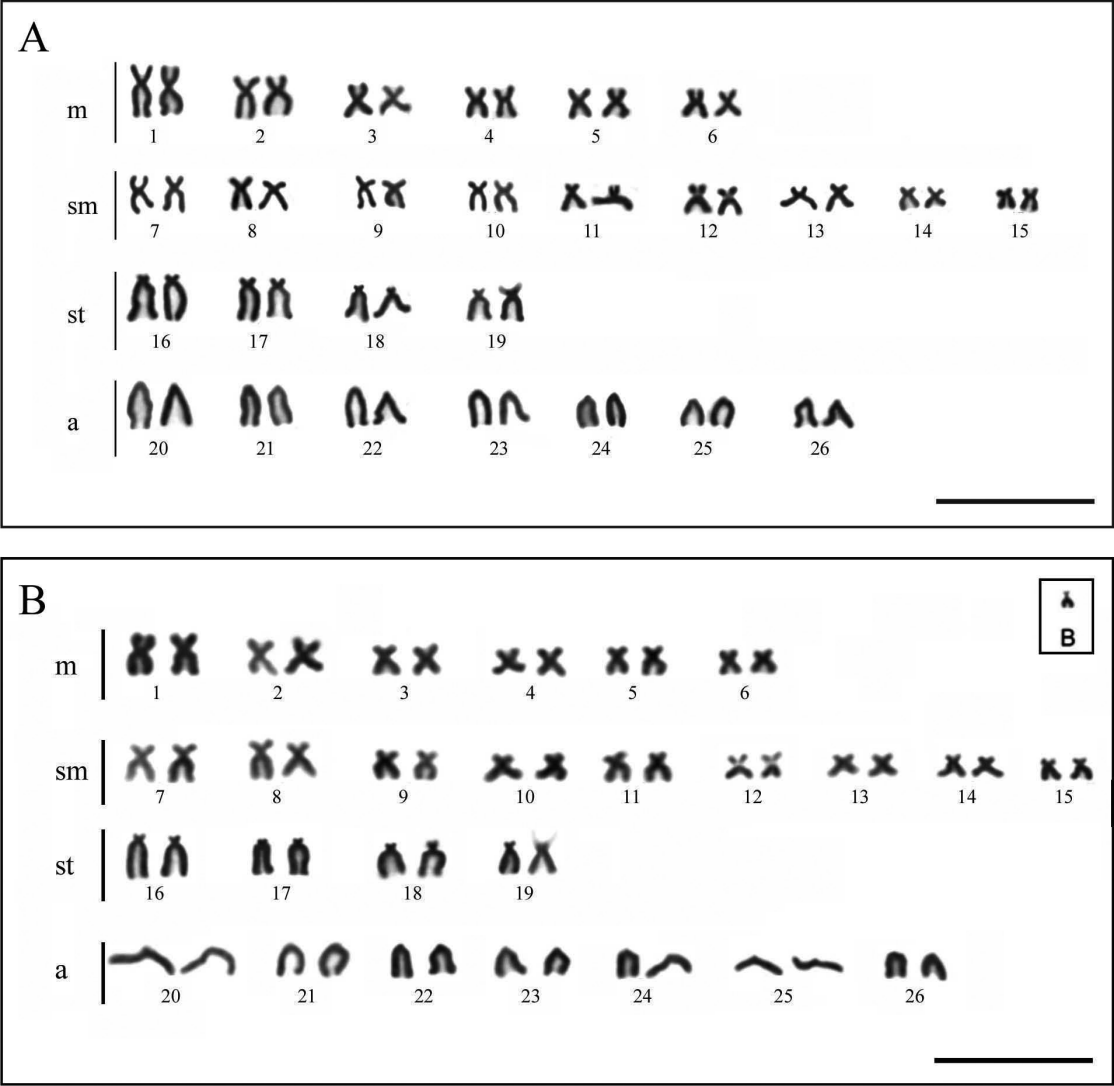
Giemsa stained chromosomes of *Hyphessobrycon eques*. **A** Karyotype without B chromosome **B** Karyotype with B chromosome. Inset show the B chromosome. Bar = 10 µm.

**Figure 2.**
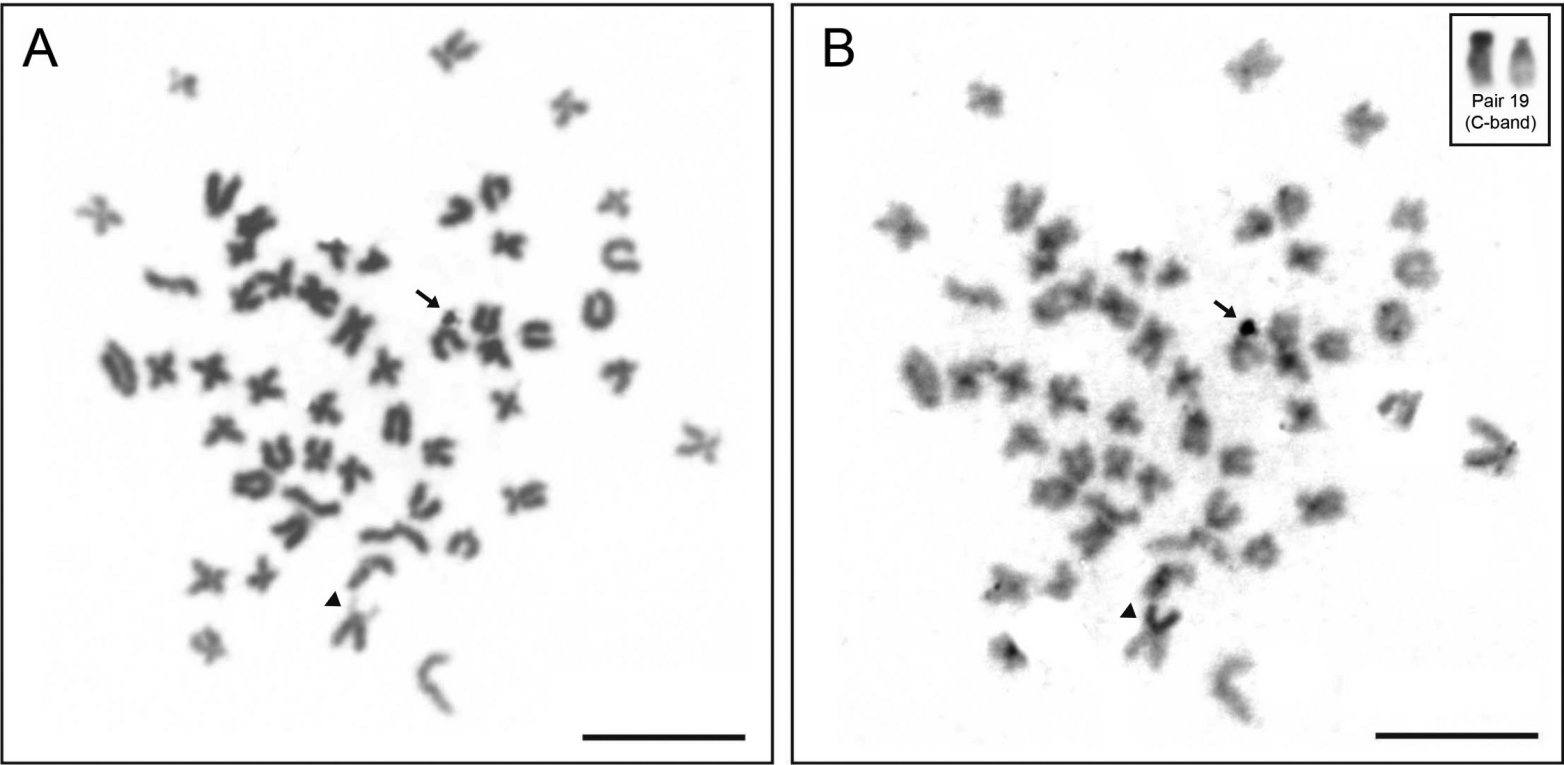
Mitotic metaphase chromosomes. **A** Giemsa stained **B** C-banding. The arrow indicates the B chromosome and the arrowhead indicates the secondary constriction. Inset show the pair 19 C-banded of an individual without B chromosome. Bar = 10 µm.

The *Hyphessobrycon eques* specimens had 2n = 52 chromosomes, and the karyotype contained 12 metacentric, 18 submetacentric, 8 subtelocentric, and 14 acrocentric chromosomes (12m + 18sm + 8st + 14a), yielding a FN of 90 for both sexes (Figure 1a, Table 1). A region of secondary constriction was evident on the short arm of one homolog of pair 19 (Figure 1a, b). One subtelocentric B chromosome was observed in all cells of two females (Figures 1b, 2a). Heterochromatic regions were observed mainly on centromeric regions, and a large block was observed in the short arm on one homolog of pair 19 (individuals with and without B chromosome) (Figure 2b). The B chromosome showed the long arm fully heterochromatic (Figure 2b).

**Table 1.** Cytogenetic data and presence of B chromosomes in the *Hyphessobrycon* genus.

Species	2*n*	Karyotype formulae	Presence of Bs	References
*Hyphessobrycon minor*	52	14m+20sm+16st	–	Arefjev (1989)
*Hyphessobrycon scholzei*	50	8m+20sm+8st+14a	–	Arefjev (1990)
*Hyphessobrycon flammeus*	52	18m/sm+32st+2a	–	Arefjev (1990)
*Hyphessobrycon herbertaxelrodi*	52	10m/sm+42st/a	–	Arefjev (1990)
*Hyphessobrycon reticulatus*	50	20m+14sm+16st/a	–	Wlasiuk and Garcia (1996)
*Hyphessobrycon bifasciatus*	50	16m+10sm+12st+12a	–	Miyazawa (1997)
Hyphessobrycon aff. santae	50	12m+10sm+10st+18a	–	Miyazawa (1997)
*Hyphessobrycon reticulatus*	50	14m+20sm+16st	–	Carvalho et al. (2002a)
*Hyphessobrycon reticulatus*	50	-	–	Carvalho et al. (2002b)
*Hyphessobrycon griemi*	48	-	–	Carvalho et al. (2002b)
*Hyphessobrycon anisitsi*	50	6m+16sm+12st+16a	–	Centofante et al. (2003)
*Hyphessobrycon anisitsi*	50	18m+10sm+6st+16a	–	Mendes et al. (2011)
*Hyphessobrycon luetkenii*	50	6m+8sm+36a	–	Mendes et al. (2011)
*Hyphessobrycon eques*	52	14m+16sm+4st+18a	–	Martinez et al. (2012)
*Hyphessobrycon eques*	52	12m+18sm+8st+14a	0–1♀/0♂	Present study

**Figure 3.**
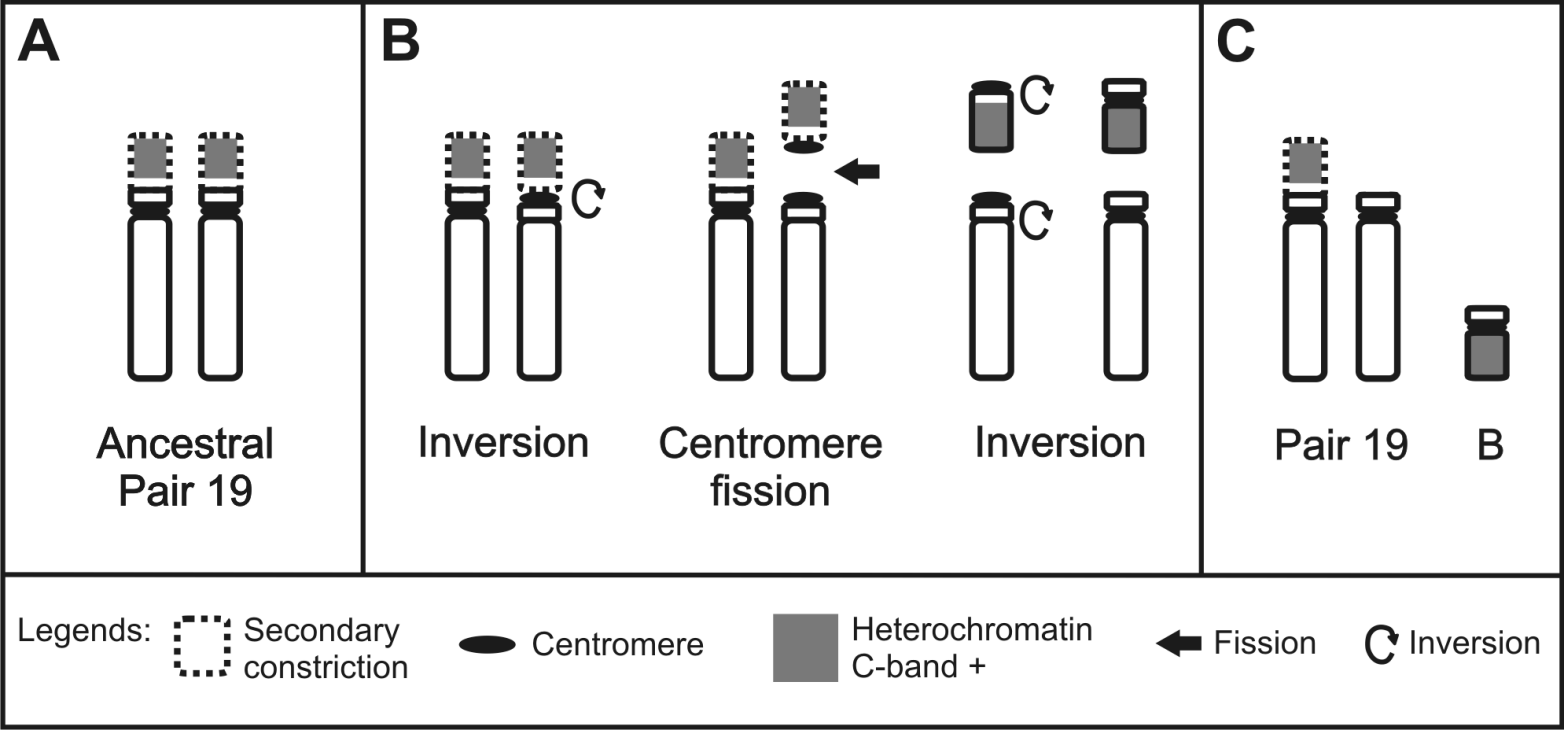
Scheme showing a possible origin of the B chromosome in *Hyphessobrycon eques*. **A** Pair 19 not fissioned **B** The short arm of one homologous underwent fission and inversion **C** One homologous of pair 19 without the secondary constriction and a B chromosome formed.

A summary diagram indicating a possible origin mechanism of the B chromosome in *Hyphessobrycon eques* by heterochromatin blocks is shown in Figure 3.

## Discussion

The heterochromatin was observed mainly in the centromeric regions on chromosomes of *Hyphessobrycon eques* in this present paper. On the other hand, Carvalho et al. (2002a) detected small heterochromatin blocks in the pericentromeric regions in all chromosomes of *Hyphessobrycon reticulatus* from Juquiá river (state of São Paulo, Brazil). Centofante et al. (2003) studied two populations of *Hyphessobrycon anisitsi* from adjacent hydrographic basins (upper Paraná river basin and Paraíba do Sul river basin) and also observed heterochromatic blocks mainly on pericentromeric regions of most chromosomes.

An interesting feature observed by C-band technique in the *Hyphessobrycon eques* was a heteromorphic block of heterochromatin always presents on short arm (pair 19) in all specimens (with and without B chromosomes), which another population of *Hyphessobrycon eques* studied by Martinez et al. (2012) not showed. Nevertheless, we believe that the B chromosome (observed in two *Hyphessobrycon eques* females) may be related with chromosomal rearrangements (see a possible mechanism in the Figure 3).

This study reported for the first time the presence of B chromosomes in the *Hyphessobrycon* genus. According to Leach et al. (2004) analyses of the molecular structure have shown that B chromosomes are subject to gene silencing, repetitive DNA accumulation and heterochromatinization. Thus, most of the heterochromatic of B chromosomes are due to the presence of chromatin characterized by a high degree of condensation during the cell cycle, and this natural condensation results from the high content of the repetitive DNA of many types, especially satellite and ribosomal DNAs (Camacho 2005).

Different postulations have been formulated to explain the independent evolution of B chromosomes in the genome of organisms that possess them. Camacho et al. (2000) claim that, subsequent to synaptic isolation of the B chromosome and regardless of their origin, processes of molecular evolution also can occur and determine a degenerate morphology for these genomic segments. Thus, the morphological and structural features would be more a reflex of molecular evolution processes than the way in which they originated. However, it appears that the supernumerary chromosomes do not present a model of common origin, i.e. they may have originated independently following different evolutionary paths.

One hypothesis proposed to explain the presence and function of the B chromosomes is the isochromosome (Vicente et al. 1996, Mestriner 2000, Silva et al. 2014). According to Sumner (2003), isochromosomes are chromosomes with two homologous arms, i.e. which are structurally and genetically equal and may be originated by different ways. The author explains that one of the hypotheses suggested for the emergence of this type of chromosome is the fusion between two identical acrocentric chromosomes, which most likely did not occur with the B chromosome in the *Hyphessobrycon eques* studied in this paper.

Nevertheless, the presence of one B chromosome in females may be less likely due to the sex chromosome system in the *Hyphessobrycon eques* (even if only one sex) than involved with possible chromosomal break. However, we cannot rule out the possibility that this occasional chromosome break, from now on, may have resulted in the maintenance of this element in the females and drive to differentiation of a sex chromosome system for *Hyphessobrycon eques*.
